# Debate: Could the litchi pericarp oligomeric procyanidins bioconverted by *Lactobacillus plantarum* increase the inhibitory capacity on advanced glycation end products?

**DOI:** 10.3389/fnut.2022.961078

**Published:** 2022-07-22

**Authors:** Nianjie Feng, Fei Tang, Chuanqin Hu, Lei Cheng, Zhejuan Lv, Yang Shen, Wei Li, Gengsheng Xiao, Hao Dong, Qian Wu

**Affiliations:** ^1^Key Laboratory of Fermentation Engineering (Ministry of Education), National “111” Center for Cellular Regulation and Molecular Pharmaceutics, Cooperative Innovation Center of Industrial Fermentation (Ministry of Education & Hubei Province), Hubei Key Laboratoy of Industrial Microbiology, Hubei University of Technology, Wuhan, China; ^2^Key Laboratory of Cleaner Production and Integrated Resource Utilization of China National Light Industry, Beijing Technology and Business University, Beijing, China; ^3^Hanyang Marketing Department, Hubei Tobacco Company, Wuhan, China; ^4^Key Laboratory of Green Processing and Intelligent Manufacturing of Lingnan Specialty Food, Ministry of Agriculture, College of Light Industry and Food Sciences, Zhongkai University of Agriculture and Engineering, Guangzhou, China

**Keywords:** litchi pericarp oligomeric procyanidins, lactic acid bacteria, advanced glycation end products, antioxidant activity, inhibition

## Abstract

Lactic acid bacteria (LAB) have already been used as fermentation strains to enhance the antioxidant capacity of polyphenols. Antioxidant capacity is one of the most important factors to inhibit advanced glycation end product (AGE) formation and could LAB increase the inhibitory capacity of procyanidins on AGEs formation? It was surprising that opposite results were obtained both in simulated food processing and gastrointestinal digestion systems. After incubation with *Lactobacillus plantarum (L. plantarum)*, litchi pericarp oligomeric procyanidins (LPOPCs) were bioconverted to several phenolic acids, which increased the antioxidant activity as expected. However, antiglycation ability and trapping carbonyl compounds capacity both weakened and it might be the primary reason for decreasing the inhibitory effect on AGE formation. Furthermore, it was found that LPOPCs incubated with *L. plantarum* inhibited the activity of digestive enzymes and thus decreased the digestibility of glycated protein. Our study systematically proposed for the first time that procyanidins bioconversion is an effective means to improve the antioxidant activity but has no remarkable promoting effect on AGEs inhibition.

## Introduction

Advanced glycation end products (AGEs), such as carboxymethyl lysine (CML) and carboxyethyl lysine (CEL), are generated by the reaction between reducing sugars and the free amino residues of proteins, lipids, or nucleic acids (1). Dietary AGEs, the main source of exogenous AGEs, are digested, absorbed, bound with the receptor for advanced glycation end products (RAGEs), and then lead to outbreaks of foodborne diseases (2). AGE accumulation has been proposed to be a risk factor for structural tissue damage, oxidative stress, and inflammation in aging and diseases (3). Similar results have frequently been reported in clinical investigations of human populations (4). Therefore, the study of AGE inhibitors could be beneficial to control the foodborne diseases.

Several recent articles have reported a significant antioxidant capacity of procyanidin, which was used as an effective AGE inhibitor *in vitro* (5). The main oligomeric procyanidins isolated from litchi pericarp were identified as A2 and epicatechin-(4β→ 8, 2β→ O → 7)-epicatechin-(4β→ 8)-epicatechin (6). They have been demonstrated to possess strong activity in inhibiting AGE formation *via* scavenging free radicals and trapping α-dicarbonyl compounds (7).

Lactic acid bacteria (LAB), such as *Lactobacillus plantarum, Enterococcus durans*, and *Weissella confuse*, have been widely used in the field of food (8–10). In addition, they have been shown to impact positively on health thereby increasing the desirability of such commodities by consumers (11).

Recently, the interaction between polyphenols and LAB has attracted the most attention. It was reported that polyphenols could be transformed into gallic acid, pyrogallol, and catechol by *L. plantarum* through galloyl-esterase, decarboxylase, benzyl alcohol dehydrogenase, and tannase (12, 13). The transformed phenolic acids are absorbed *in vivo* more easily than procyanidins, due to their low molecular weight and strong polarity (14–16). There is no doubt that transformed phenolic acids should possess higher antioxidant capacity and bioavailability; however, their inhibition effect on AGE formation has rarely been reported.

**Scheme 1:**
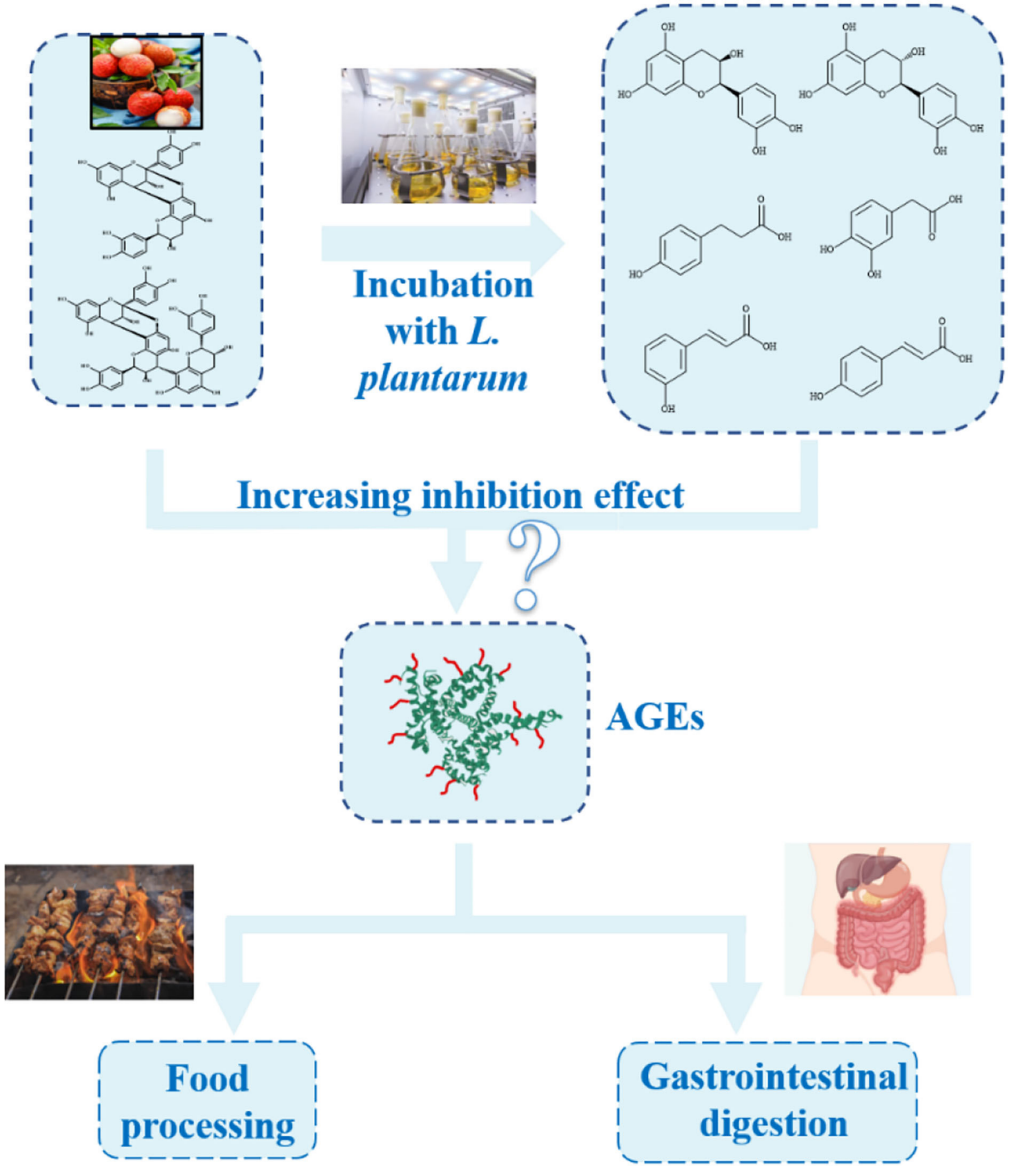
Advanced glycation end product (AGE) inhibitory effect of procyanidins in the presence of LAB both in simulated food processing and gastrointestinal digestion systems.

It is the first time to investigate the AGE inhibitory effect of procyanidins in the presence of LAB (Scheme 1). The simulated food processing and gastrointestinal digestion systems were used, and the interactions between procyanidins and LAB were investigated in order to provide a new insight into AGE inhibitors.

## Materials and methods

### Chemicals and materials

Fruits of litchi (*L. Chinensis* Guiwei) were obtained from Guangzhou, China and arrived in the laboratory within 24 h postharvest. The fruits were peeled immediately, and the pericarp was kept in sealed polyethylene bags at −18°C prior to extraction for 3 days. *L. plantarum* used in this study was purchased from the China Center of Industrial Culture Collection. (–)-Epicatechin (EC), (+)-catechin (CC), 3,4-Dihydroxyphenylacetic acid (3,4-HPLA), 4-hydroxyphenylpropionic acid (4-HPA), *m*-coumaric acid (*m*-CA), *p*-coumaric acid (*p*-CA), acetonitrile, methanol, and acetic acid were from Sigma Chemical Co. (St. Louis, MO). Formic acid and 2,4,6-tris(2-pyridyl)-s-triazine were obtained from Aladdin (Shanghai, China). CML was purchased from Toronto Research Chemicals Inc. (Toronto, Ontario, Canada). All other chemicals were of analytical grade and purchased from Sinopharm Chemical Reagent Co., Ltd. of China (Beijing, China).

### Extraction of oligomeric procyanidins from litchi pericarp

The oligomeric procyanidins of litchi pericarp (LPOPCs) were obtained according to the method by Li et al. (17). Briefly, frozen litchi pericarp fragments were extracted by 70% ethanol, eluted by 70% ethanol, and subsequently extracted by ethyl acetate.

### Growth experiments

*Lactobacillus plantarum* was inoculated in de Man, Rogosa and Sharpe (MRS) medium at 37°C for 18 h, then diluted to obtain the concentration of approximately 10^7^ cfu/ml. The bacterial suspension was mixed with LPOPCs at different concentrations and maintained at 37°C for 48 h under aerobic conditions. The growth was measured at 2 h intervals by assessing optical density at 600 nm (OD600) using a UV-1601 spectrophotometer (Ulupure Instrument and Equipment Co, Xian, China).

### Simulated systems

#### Simulated food processing system

Glucose (42 mM), lysine (42 mM), and sodium azide (3 mM) were used as a glucose-lysine model heating at 50°C for 5 days.

#### Simulated gastrointestinal digestion system *in vitro*

Glycated bovine serum albumin (BSA) was prepared using the method described by Zhao et al. (2). Glycated BSA of 14 mg and 2 ml of inoculated LPOPC were added to 8 ml of simulated gastric fluid (SGF, 13.2 mg pepsin mixed with 50 ml of 0.6 M NaCl at pH 2). After 2 h of incubation at 37°C, pH of the SGF was adjusted to 7 with 1 M NaHCO_3_ and thereupon mixed with 2 ml of simulated intestinal fluid (SIF, 2 g/L pancreatin and 12 g/L bile salt). The intestinal digestion continued *in vitro* for 2 h at 37°C and was quenched in boiling water bath for 3 min.

#### Determination of fluorescent AGEs and CML

Fluorescent AGEs and CML were determined according to methods by Wu et al. (18). *L. plantarum* and non-inoculated LPOPCs were used as control.

### Analysis of LPOPCs metabolites after *L. plantarum* bioconversion

A liquid chromatography/electrospray ionization with multi-stage mass spectrometry (LC-ESI-MSn, Agilent Technologies Co., Ltd., Santa Clara, CA, USA) was used to detect the composition and structure of phenolic acids after fermentation (17). A ZORBAX Eclipse XDB-C18 column (150 × 4.6 mm, 5 μm particle size, Shimadzu Co., Kyoto, Japan) was prepared with the column temperature set at 30°C. The mobile phases were (A) 0.4% (v/v) aqueous acetic acid and (B) acetonitrile. Elution conditions were as follows: a linear gradient from 5 to 35% B in 40 min, from 35 to 50% B in 5 min, from 50 to 80% B in 5 min, and from 80 to 5% B in 5 min, at a flow rate of 1.0 ml/min. In a negative-ion mode, an ESI mass spectrometer in the range of 100–1,200 m/z was recorded. Other conditions were as follows: orifice voltage, −30 V; spray voltage, 4 kV; gas flow rate, 20 ml/min; and heat capillary temperature, 325°C.

### Ferric reducing antioxidant power (FRAP) assay

The assay was based on the reduction of a ferric 2,4,6-tris(2-pyridyl)-1,3,5-triazine (Fe^3+^-2,4,6-tripyridyl-s-triazine [TPTZ]) to the ferrous form (Fe^2+^-TPTZ), followed by spectrophotometric analysis (19). Briefly, the FRAP reagent was prepared by mixing 10 volumes of 300 mmol/L acetate buffer, pH 3.6, with 1 volume of 10 mmol/L TPTZ in 40 mmol/L hydrochloric acid, and with 1 volume of 20 mmol/L ferric chloride. In total, 50 μl of sample were added to 1.5 ml of the FRAP reagent. The absorbance of the reaction mixture was then recorded at 593 nm after 4 min. Aqueous solutions of known Fe^2+^ concentration, in the range of 100–2,000 μmol/L (FeSO_4_ + 7H_2_O) were used for calibration.

### Evaluation of glyoxal (GO) trapping capacity

The GO trapping capacity was tested using the method described by Peng et al. (20) with some modifications. PD (derivatization agent, 20 mM) was freshly prepared in PBS (pH 7.4, 0.02 M). The sample was mixed with the peritoneal dialysis (PD) solution in the ratio of 1:1 and maintained at 37°C for 0.5 h. The derivative product was then injected into an high-performance liquid chromatography (HPLC) system equipped with an LC-20AD liquid chromatograph (Shimadzu, Japan) and a Diamonsil TM-C18 column (4.6 × 200 mm, 5 μm, Shimadzu Co., Kyoto, Japan).

### Evaluation of particle size

The digested solution was diluted to 1 mg/ml using 25 mmol/L phosphate buffer and filtered through a 0.22 μm membrane. The particle size was achieved using a Nano Zsp-MPT-2 System (Malvern Instruments Inc., Worcestershire, UK) and measured at a fixed 173° scattering angle in 3.5 ml of plastic cuvettes.

### Digestive enzyme activity inhibition ability test

#### Pepsin activity assay

The enzyme activity was detected by the method of Zhu et al. (21). The incubation mixture contained 30 units of pepsin, 10 mM HCl, and variable amounts of bovine hemoglobin in the test tube. The assay was conducted in the presence of LPOPCs at different concentrations. After 10 min of incubation at 37°C, 0.7 ml of 10% trichloroacetic acid was added to precipitate hemoglobin. After centrifugation, the supernatant was determined by recording the absorbance at 280 nm.

#### Trypsin activity assay

Trypsin activity was assayed by utilizing N-Benzoyl-DL-arginine-4-nitroanilide hydrochloride (BAPNA) as substrate (22). In the study, BAPNA solution was injected into the digested solution. Then, the mixture was incubated with the diluted enzyme at 37°C for appropriate time intervals. The reaction was stopped by the addition of 30% acetic acid for the determination of the absorbance at 410 nm. The BAPNA solution directly incubated with diluted enzyme was used as a control.

### Statistical data analysis

All experiments were performed in triplicate and reported as mean ± standard deviation (SD). Significant differences between mean values were determined by one-way ANOVA followed by Duncan's multiple range tests and confirmed the results at 95% confidence interval (CI, *p* < 0.05). The graph was drawn by OriginPro 8.0.

## Results and discussion

### Effect of LPOPCs on the growths of *L. plantarum*

**Figure 1:**
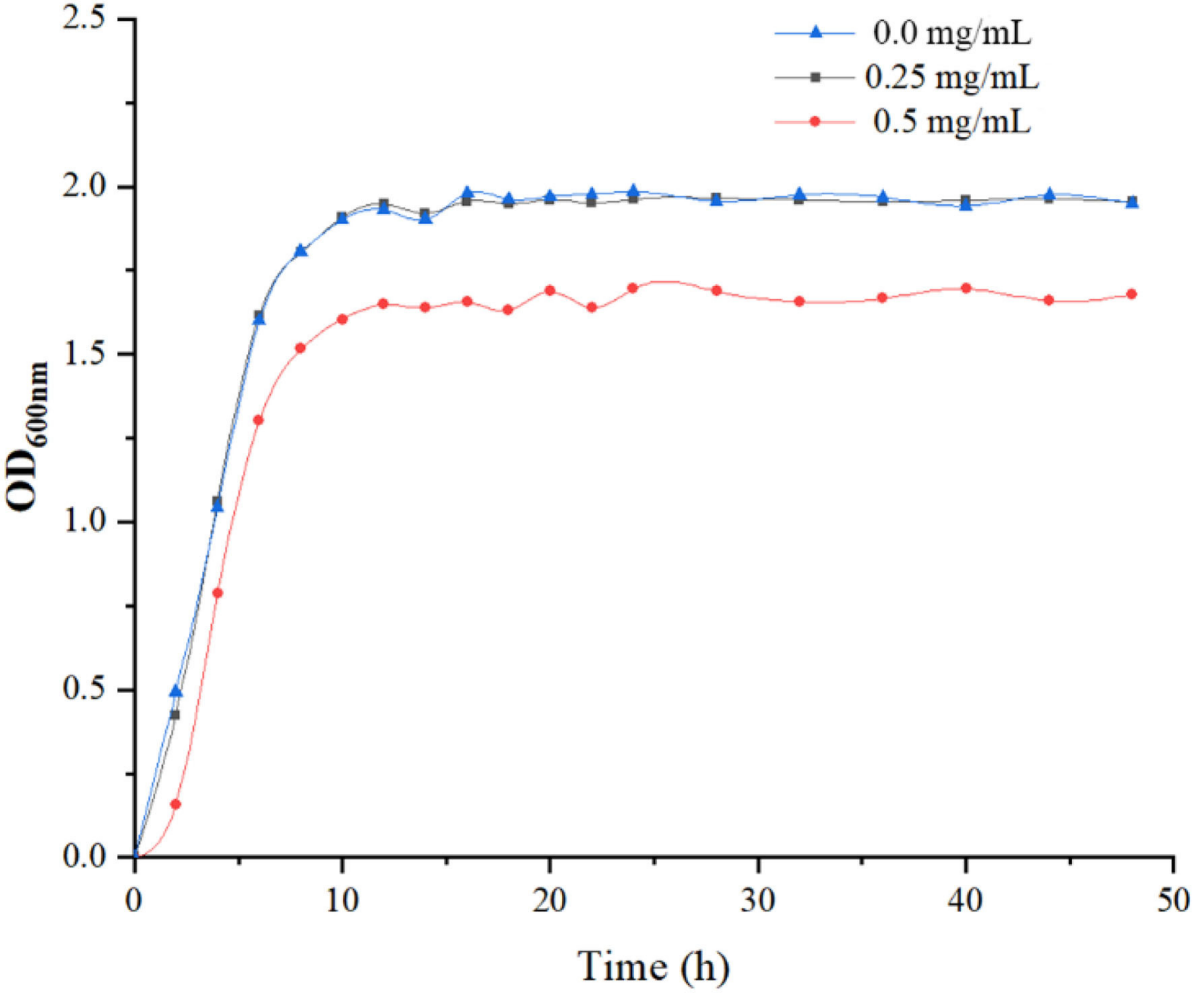
Growth curve of *L. plantarum* in de Man, Rogosa and Sharpe (MRS) medium at 37°C.

The growth of *L. plantarum* was significantly inhibited by LPOPCs at a concentration of 0.5 mg/ml. It has been found that the high concentration of antioxidant compounds, particularly of polyphenols, negatively affected the LAB wall and membrane integrity, dissipated the pH gradient, and delayed the metabolism of carbohydrates (23). Whereas, as seen in Figure 1, no obvious change is found at a concentration of 0.25 mg/ml. Thus, 0.25 mg/ml of LPOPCs would be used for follow-up research.

### Inhibition effect of inoculated LPOPCs on AGEs formation

#### The fluorescent AGEs

**Figure 2:**
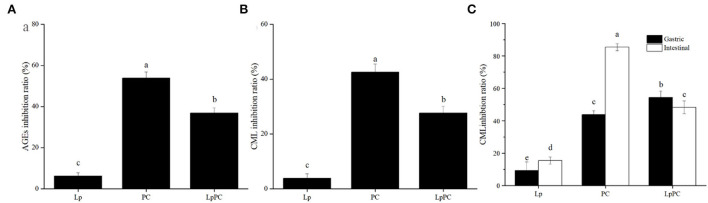
Inhibition ratios of inoculated litchi pericarp oligomeric procyanidins (LPOPCs) on fluorescent advanced glycation end product (AGEs) and carboxymethyl lysine (CML) formation. Fluorescent AGEs **(A)**, CML in simulated food processing system **(B)**, and CML in gastrointestinal digestion system *in vitro* **(C)**. LP, *L. plantarum* group; PC, non-inoculated LPOPCs group; LpPC, inoculated LPOPCs group.

The inhibitory ratio of inoculated LPOPCs on fluorescent AGEs was increased as compared to *L. plantarum* alone, reaching 36.9% (Figure 2A). It was interesting that it showed a stronger inhibitory effect on the non-inoculated LPOPCs, reaching 53.8%. There was no doubt that the fermentation process had a significant impact on the AGE inhibitory capacity of LPOPCs.

#### CML

Carboxymethyl lysine was the most widely typical AGEs, which has been selected as the main marker of AGEs in many research studies (24). In the simulated food processing system, it was found that the inhibitory ratio of inoculated LPOPCs group can reach 27.7% (Figure 2B). While the inhibition ratio of non-inoculated LPOPCs could reach to 42.5%, which was consistent with fluorescent AGE inhibition effect as above.

For the simulated gastric digestion system *in vitro*, the inoculated LPOPCs resulted in an improving inhibitory effect as shown by increasing the inhibition ratio from 9.2 to 54.3% (Figure 2C, solid). However, for the simulated intestinal digestion system *in vitro*, non-inoculated LPOPCs hold a relatively high inhibition ratio, reaching almost 85.5%. Compared with the gastric digestion stage, LPOPCs had a much better inhibitory effect on AGE formation. One reason might be that phenolic compounds showed better bioaccessibility in the intestinal environment (25). Another reason was that glycated protein was resolved into glycated peptides with more binding site after gastrointestinal digestion (26). Overall, our study found that non-inoculated LPOPCs had a better inhibitory effect on AGE formation both in simulated food processing and gastrointestinal digestion systems. Next, we have explored the mechanism.

### Identification of bioconversion products of the inoculated LPOPCs

**Figure 3:**
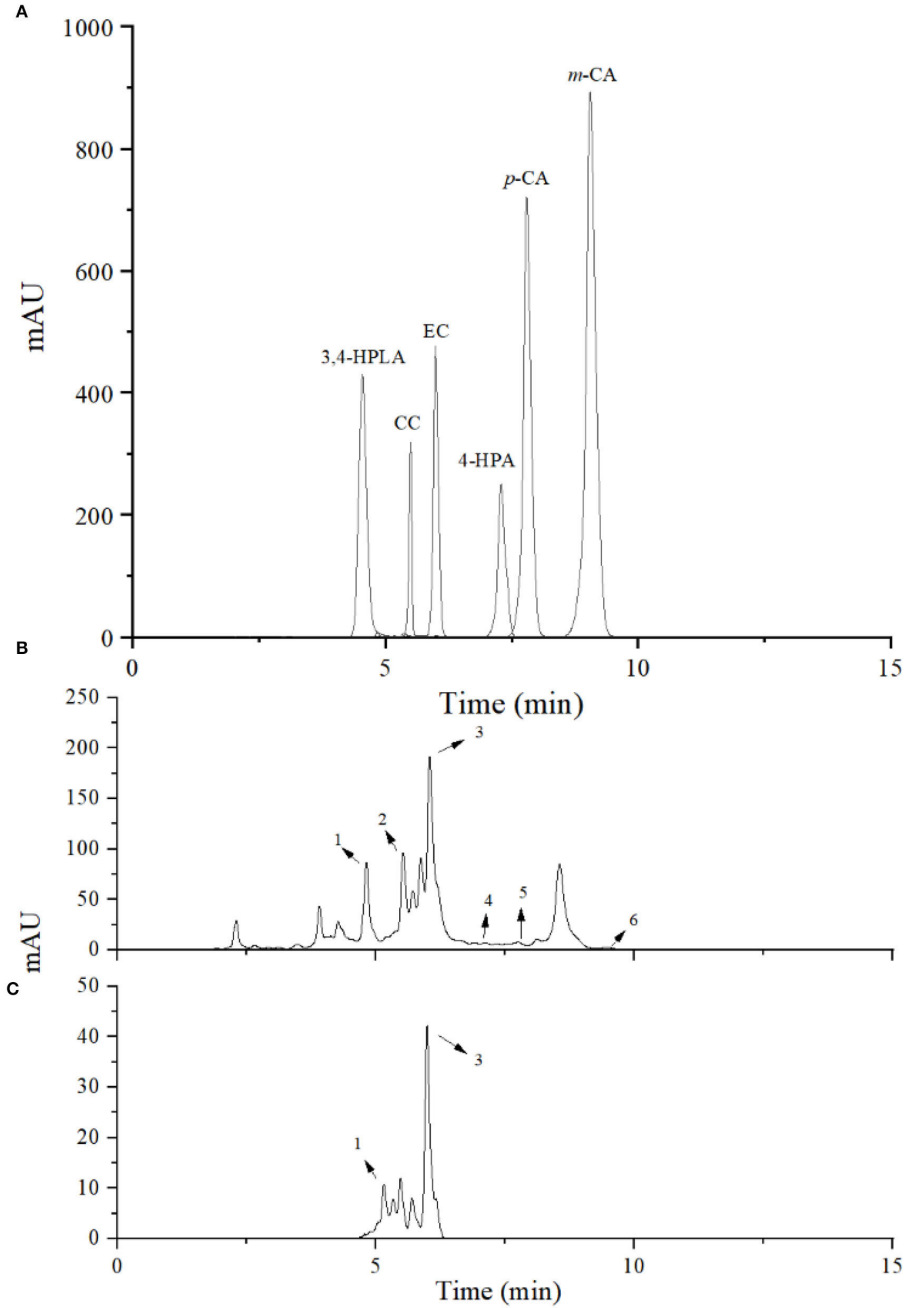
High-performance liquid chromatography (HPLC) chromatogram of bioconversion products. Standard phenolic acids **(A)**, litchi pericarp oligomeric procyanidins (LPOPCs) after incubation with *L. plantarum* **(B)**, and LPOPCs **(C)**. Peaks 1–6 were 3,4-HPLA, CC, EC, 4-HPA, *p*-CA, and *m*-CA.

Bioconversion products of the inoculated LPOPCs were analyzed by HPLC-MS^2^ (Figure 3). Six compounds (3,4-HPLA, 4-HPA, *m*-CA, *p*-CA, CC, and EC) are listed in Table 1, based on the accurate masses of the parent ion, product ions, and retention time. In total, 73.7% of CC and 82.2% of EC were decreased after fermentation, and some of them were bioconverted to phenolic acids, such as 3,4-HPLA, 4-HPA, *m*-CA, and *p*-CA. LPOPCs could be bioconverted by *L. plantarum* to the small molecules by some inducible enzymes, such as tannase, which possess the ability to hydrolyze the ester and depside linkages in wide-ranging substrates, such as gallotannins, epigallocatechin-3-gallate, gallic acid esters, and EC gallate, to release gallic acid and glucose (27, 28). Meantime, probably a series of other reactions, such as demethylation, dehydroxylation, and decarboxylation, occurred during the bioconversion process (12). The bioconversion products possessed less molecular weight and stronger polarity than their original, which would increase their bioavailability *in vivo* (29). Thus, the kinds and concentrations of phenolic acids in fermentation liquor and the significant decrease in CC and EC may be closely related to the AGEs inhibitory effect.

**Table 1:** Bioconversion products of litchi pericarp oligomeric procyanidins (LPOPCs) inoculated by *L. plantarum*.

**Compounds**	**Retention time (min)**	**Parent ion (m/z)**	**Product ion (m/z)**	**Concentrations (μg/mL)**	
				**0 h**	**40 h**
3,4-HPLA	3.11	167.03	163.09	ND	2.6 ± 0.2
4-HPA	3.93	165.06	147.58	ND	1.9 ± 0.2
*m*-CA	4.36	163.04	119.05	ND	0.5 ± 0.0
*p*-CA	4.08	163.04	119.05	ND	0.6 ± 0.4
CC	3.26	288.92	244.74	56.3 ± 1.0	14.8 ± 2.3
EC	3.53	288.92	244.83	53.8 ± 0.7	9.6 ± 1.3

### Evaluation of antioxidant capacity

**Figure 4:**
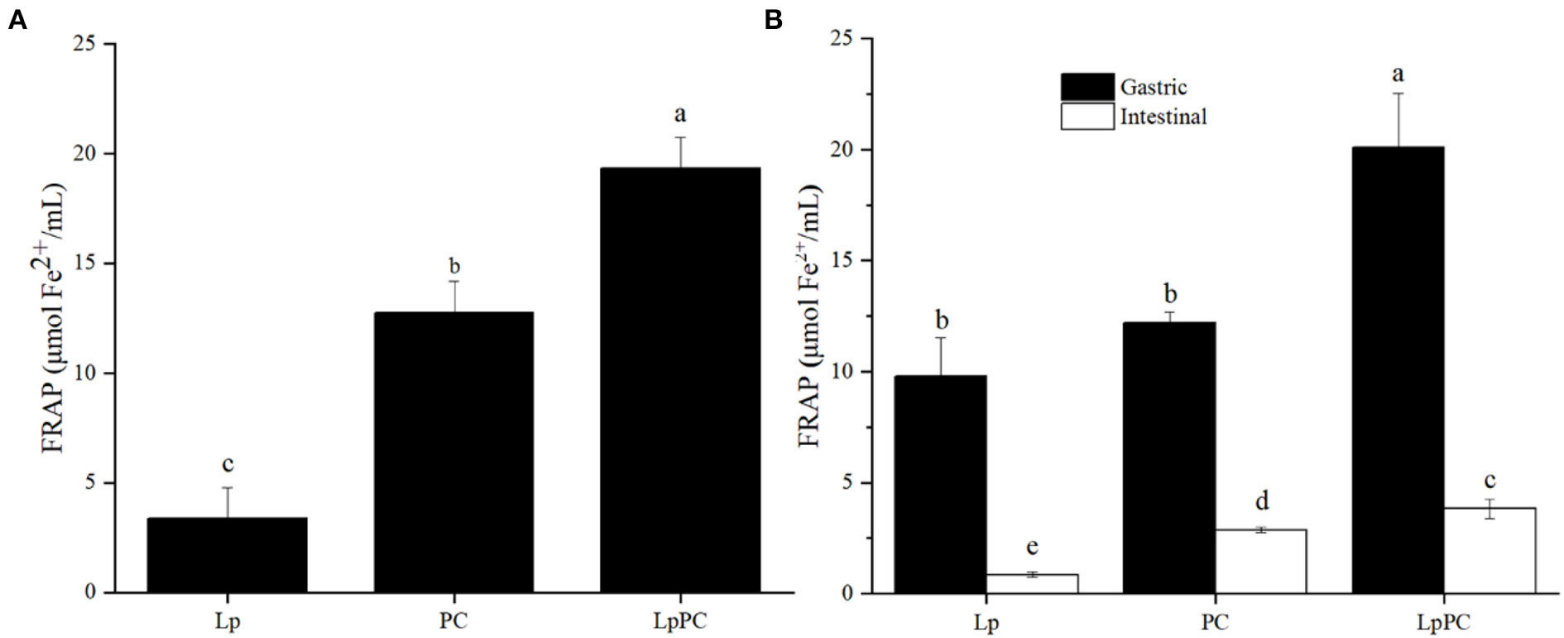
Antioxidant activity of inoculated litchi pericarp oligomeric procyanidins (LPOPCs) in simulated food processing system **(A)** and gastrointestinal digestion system *in vitro* **(B)**.

For the simulated food processing system, the fermentation liquor of *L. plantarum* showed little antioxidant activity (3.4 μmol Fe^2+^/ml by FRAP, Figure 4A). While, the FRAP of LPOPCs after fermentation increased from 12.7 μmol Fe^2+^/ml to 19.3 μmol Fe^2+^/ml. It had been reported that procyanidins could be degraded by LAB into some phenolic acids that process strong antioxidant capacities (17).

For the gastric digestion system *in vitro*, the antioxidant activity of the inoculated LPOPC was significantly enhanced and reached to 20.1 μmol Fe^2+^/ml by FRAP (Figure 4B). However, the FRAP of the inoculated LPOPCs was only 3.8 μmol Fe^2+^/ml in the intestinal digestion stage. It was because that the intestinal digestion stage was not an ideal acid environment for phenolic compounds with higher antioxidant capacity (30). Another main reason was that some active LPOPCs had already been mingled and connected with glycated protein in gastric digestion (31).

### Evaluation of GO trapping capacity

**Figure 5:**
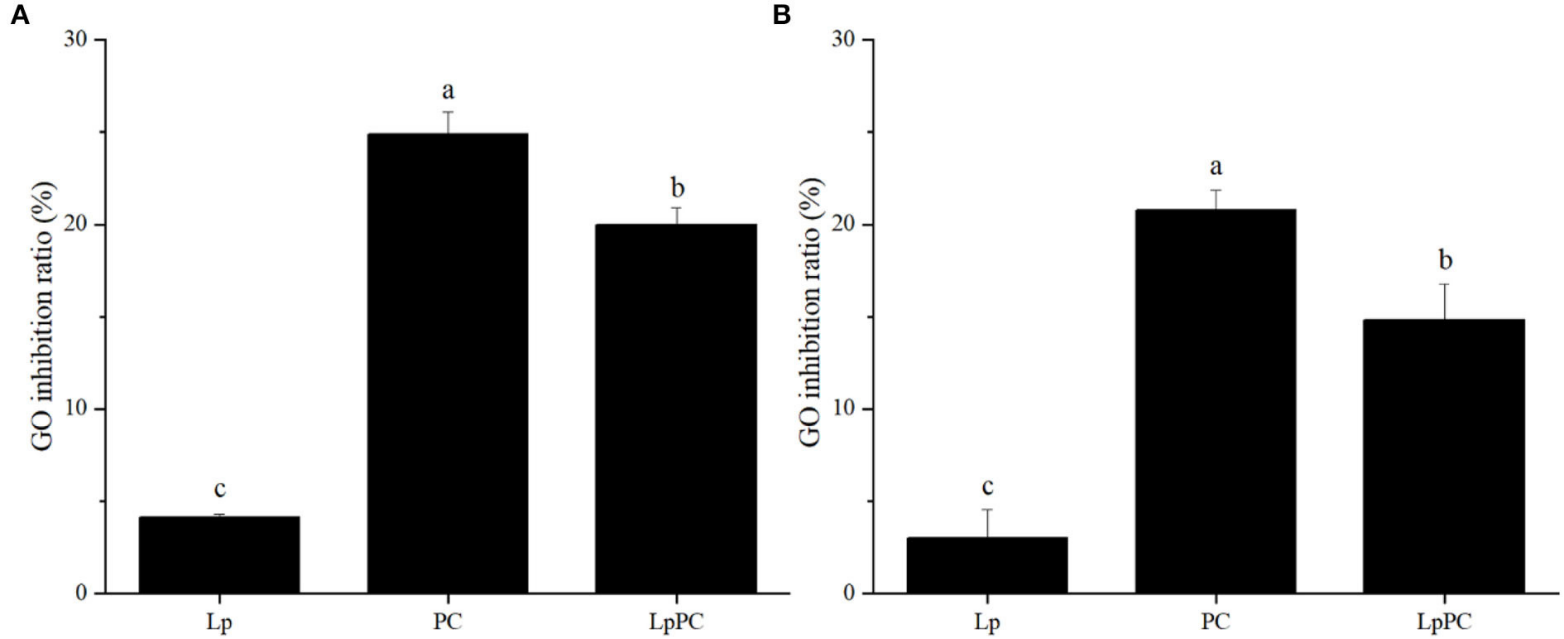
Glyoxal (GO) inhibition ratios of inoculated litchi pericarp oligomeric procyanidins (LPOPCs) in simulated food processing system **(A)** and intestinal digestion system *in vitro* **(B)**.

Glyoxal, a typical α-dicarbonyl compound, is the primary intermediate that forms AGEs in both food processing and gastrointestinal digestion systems (3, 4). LPOPCs were composed of a variety of oligomeric procyanidins, which could be attached to α-dicarbonyl compounds *via* electrophilic substitution to form adducts (32). In the simulated food processing system, 24.9% of GO was effectively trapped by LPOPCs (Figure 5A). Inoculated LPOPCs showed a lower GO inhibition ratio, only reaching 19.5%. It might be that LPOPCs were bioconverted to some invalid molecular fragments. The GO inhibition ratio in the gastric digestion was not provided, because it was found that procyanidins hardly combined with α-dicarbonyl compounds at low pH (pH ≤ 4) (25). At the intestinal digestion stage, the GO inhibition ratios were 20.8% for LSOPCs and only 14.8% for inoculated LPOPCs (Figure 5B).

### Effect of inoculated LPOPCs on glycated BSA digestion

**Figure 6:**
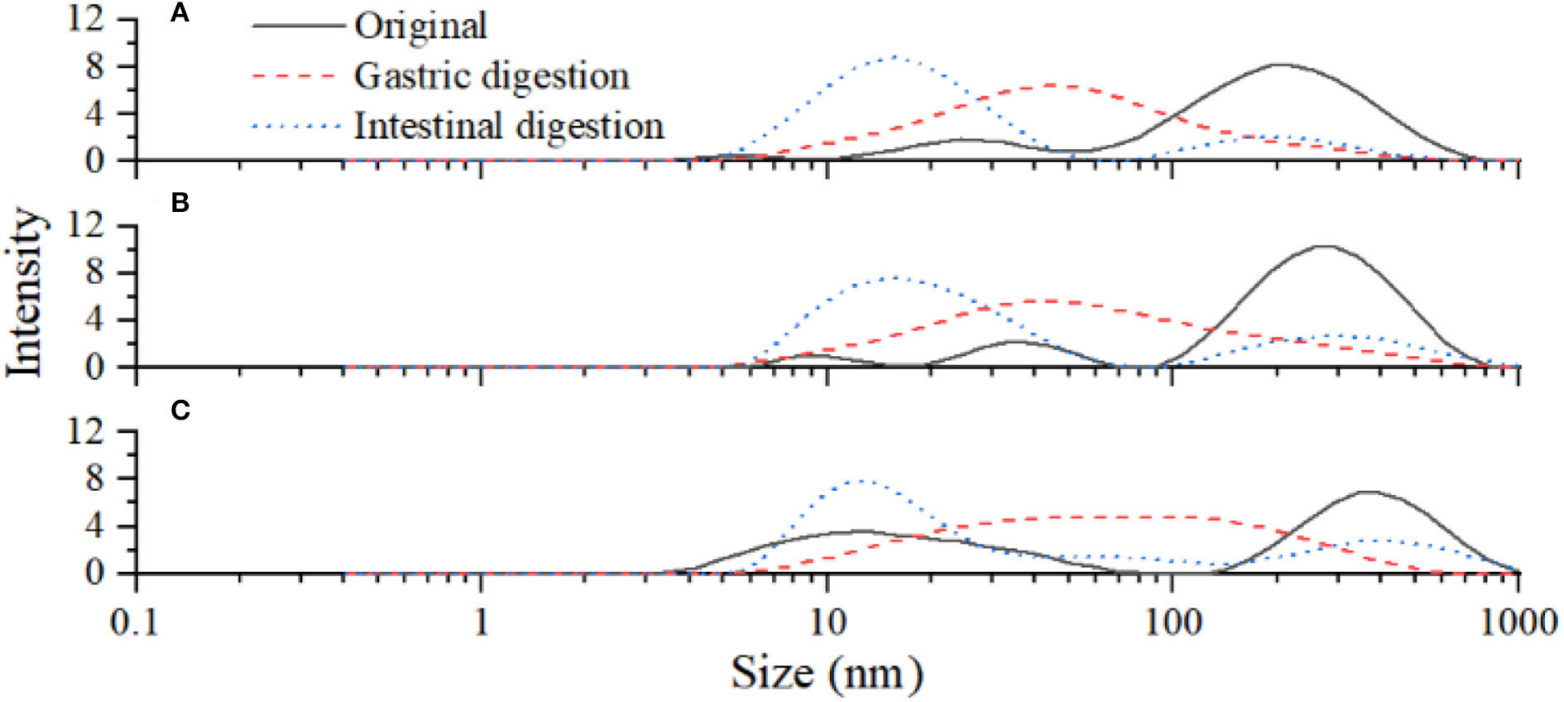
Particle size distribution of glycated bovine serum albumin (BSA) after gastrointestinal digestion. *L. plantarum* group **(A)**, non-inoculated litchi pericarp oligomeric procyanidins (LPOPCs) group **(B)**, and inoculated LPOPCs group **(C)**.

Glycated BSA was digested *in vitro* by protease and the changes in protein particle size are shown in Table 2. After gastrointestinal digestion, the particle size peaks of all samples were gradually moved to the left (Figure 6). The average particle sizes of *L. plantarum* and non-inoculated LPOPCs groups were decreased from 113.6 to 18.8 nm and from 116.8 to 29.1 nm, respectively. It was indicated that glycated BSA was hydrolyzed to some small molecules, such as peptides and amino acids. The average particle size of the inoculated LPOPCs group also decreased, however, higher particle size seemed to be found in gastrointestinal digestion processes.

**Table 2:** Particle sizes distribution of glycated BSA after gastrointestinal digestion.

**Samples**	**Particle size (nm)**		
	**Original**	**Gastric digestion**	**Intestinal digestion**
Lp	113.6 ± 7.9	39.7 ± 3.3	18.8 ± 2.3
PC	116.8 ± 5.2	45.1 ± 3.3	29.1 ± 1.7
LpPC	109.8 ± 8.6	61.1 ± 5.7	39.4 ± 3.2

^a^LP, *L. plantarum group*; PC, non-inoculated LPOPCs group; LpPC, inoculated LPOPCs group.

### Inhibitory effect of inoculated LPOPCs on pepsin and trypsin activities

**Figure 7:**
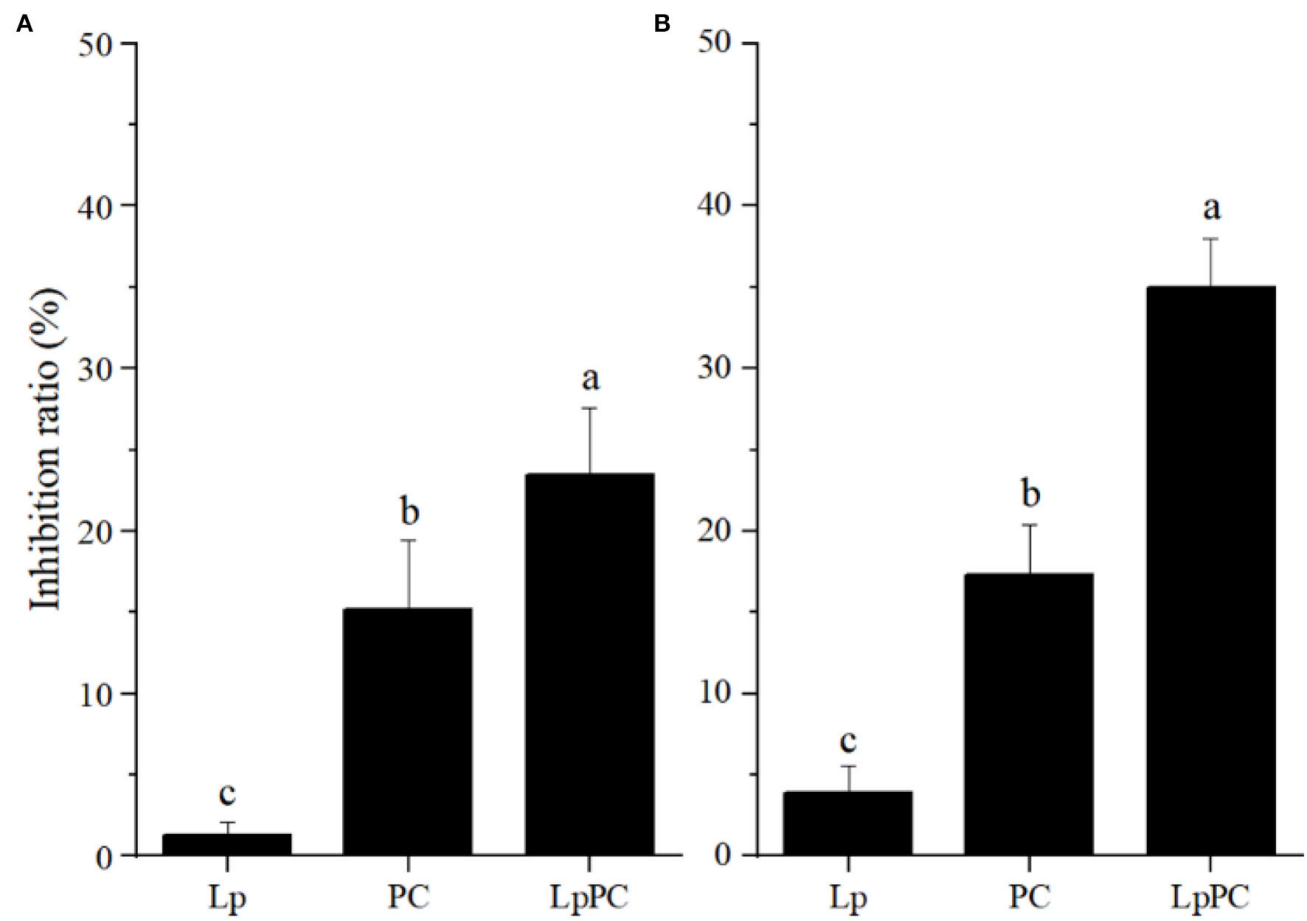
Inhibitory effect of inoculated litchi pericarp oligomeric procyanidins (LPOPCs) on pepsin **(A)** and trypsin **(B)** activities.

It was reported that polyphenols could affect protein digestibility *in vivo* (33). The combination of polyphenols and protein can shield the target sites of enzymes and subsequently resulted in a decrease of enzyme activities (22, 34). The inhibitory effects of inoculated LPOPCs on pepsin and trypsin activities are shown in Figure 7. The inoculated LPOPCs group presented the strongest inhibition effect on pepsin and trypsin activities. In addition, the inhibition ratios reached 23.5 and 35.0%, respectively. The primary reason might be that phenolic acids formed by catabolism of procyanidins had higher bioactivity than their parents (35).

### Comprehensive performance evaluation of bioconversion products

The antioxidant activity of the LPOPCs after incubation with *L. plantarum* was enhanced, however, the antiglycation capacity was weakened. In order to investigate the mechanism, the antiglycation, antioxidant, and α-dicarbonyl compounds scavenging capacity of bioconversion products of inoculated LPOPCs were determined. 3,4-HPLA is a suitable radical scavenger (36) and showed a 56.7% of CML inhibition rate. However, the bioconversion products (3,4-HPLA, 4-HPA, *m*-CA, *p*-CA) all had lower antiglycation, antioxidant, and α-dicarbonyl compound scavenging capacities (Table 3). It might be the main reason why the fermentation process had a negative impact on the inhibition effect of LPOPCs on fluorescent AGE and CML formation.

**Table 3:** Antiglycation, antioxidant, and α-dicarbonyl compound scavenging capacities of bioconversion products.

**Compounds**	**Fluorescent AGEs inhibition rate (%)**	**CML inhibition rate (%)**	**GO scavenging capacity (%)**	**FARP (μmol Fe^2+^/mL)**
3,4-HPLA	33.2 ± 3.7^a^	56.7 ± 3.1^a^	13.5 ± 1.0^c^	2.9 ± 0.1^b^
4-HPA	17.1 ± 2.4^b^	38.3 ± 3.2^bc^	11.5 ± 0.8^c^	0.5 ± 0.1^e^
*m*-CA	11.9 ± 5.8^bc^	14.7 ± 2.5^d^	8.7 ± 0.1^d^	1.0 ± 0.2^d^
*p*-CA	7.3 ± 2.1^c^	16.7 ± 3.1^d^	6.9 ± 1.3^d^	0.4 ± 0.1^e^
CC	63.7 ± 4.5^a^	33.7 ± 3.2^c^	29.8 ± 1.7^b^	3.8 ± 0.3^a^
EC	52.5 ± 2.0^a^	40.3 ± 5.1^b^	24.2 ± 1.7^a^	2.5 ± 0.4^c^

Simple correlation analyses were performed using Dunnett's test and then labeled with different superscript lowercase letters.

a The data were given as mean ± SD (*n* = 3). Different letters indicated a significant difference (*p* < 0.05).

## Conclusion

Antioxidant activity of LPOPCs was obviously increased after incubation with *L. plantarum*, but the AGEs inhibition effect was decreased both in simulated food processing and gastrointestinal digestion systems *in vitro*. The bioconversion products of the inoculated LPOPCs were identified by HPLC-MS^2^ subsequently. Bioconversion products had lower antiglycation and α-dicarbonyl compound scavenging capacities might be the main reason of decrease in AGE inhibition effect of inoculated LPOPCs. In addition, the digestibility of glycated protein was inhibited by inoculated LPOPCs. Our results suggested that procyanidins bioconversion was an effective means to improve antioxidant activity, but had no significant promoting effect on AGE inhibition.

## Data availability statement

The original contributions presented in the study are included in the article/supplementary material, further inquiries can be directed to the corresponding author/s.

## Author contributions

NF: conceptualization, methodology, investigation, and writing-original draft. FT: data curation and writing-original draft. CH and YS: data curation. LC and ZL: methodology. WL: methodology and investigation. GX: supervision. HD: supervision and funding acquisition. QW: writing-review and editing, supervision, and funding acquisition. All authors contributed to the article and approved the submitted version.

## Funding

This work was financially supported by the National Natural Science Foundation of China (Nos. 32001705, 21908048, 31901808, and 31901643), the Collaborative Grant-in-Aid of the HBUT National 111 Center for Cellular Regulation and Molecular Pharmaceutics (No. XBTK-2021003), Key Laboratory of Green Processing and Intelligent Manufacturing of Lingnan Specialty Food, Ministry of Agriculture, and the Open Research Fund Program of Key Laboratory of Cleaner Production and Integrated Resource Utilization of China National Light Industry (CP-2020-YB6).

## Conflict of interest

The authors declare that the research was conducted in the absence of any commercial or financial relationships that could be construed as a potential conflict of interest.

## Publisher's note

All claims expressed in this article are solely those of the authors and do not necessarily represent those of their affiliated organizations, or those of the publisher, the editors and the reviewers. Any product that may be evaluated in this article, or claim that may be made by its manufacturer, is not guaranteed or endorsed by the publisher.
